# Peripheral nervous system manifestations of Shiga toxin-producing *E. coli*-induced haemolytic uremic syndrome in children

**DOI:** 10.1186/s13052-021-01133-1

**Published:** 2021-09-06

**Authors:** Luisa Santangelo, Giuseppe Stefano Netti, Diletta Domenica Torres, Giovanni Piscopo, Vincenza Carbone, Luciana Losito, Leonardo Milella, Maria Luigia Lasorella, Pasquale Conti, Delio Gagliardi, Maria Chironna, Federica Spadaccino, Elena Bresin, Antonio Trabacca, Elena Ranieri, Mario Giordano

**Affiliations:** 1 Pediatric Nephrology and Dialysis Unit, Pediatric Hospital ‘Giovanni XXIII’, Bari, Italy; 2 Department of Medical and Surgical Sciences, Clinical Pathology Unit and Center for Molecular Medicine, University of Foggia, Viale Luigi Pinto -, 71122 Foggia, Italy; 3 Scientific Institute I.R.C.C.S. “E. Medea”- Unit for Severe disabilities in developmental age and young adults (Developmental Neurology and Neurorehabilitation), Brindisi, Italy; 4 Intensive Care Unit, Pediatric Hospital “Giovanni XXIII”, Bari, Italy; 5 Pediatric Neurology Unit, Pediatric Hospital “Giovanni XXIII”, Bari, Italy; 6 Department of Biomedical Sciences and Human Oncology, Hygiene Unit, University of Bari “Aldo Moro”, Bari, Italy; 7 Clinical Research Center for Rare Diseases ‘Aldo e Cele Daccò’, Istituto di Ricerche Farmacologiche Mario Negri IRCCS, Bergamo, Italy

**Keywords:** Hemolytic uremic syndrome, Enterohemorrhagic *Escherichia Coli*, Peripheral nervous system, Eculizumab, Plasma exchange, Neurorehabilitation

## Abstract

**Background:**

The Neurological involvement is the most common extra-renal complication of Shiga toxin-producing *E. coli*-hemolytic uremic syndrome (HUS) or typical HUS. On brain magnetic resonance examination, main neurological signs encompass acute lesions of the basal ganglia and the white matter, which could usually regress after Eculizumab infusion. In contrast, peripheral nervous system (PNS) manifestations in typical HUS are very rare and, when occurring, they require a careful management of neurological sequelae and an intensive multidisciplinary neuro-rehabilitation program.

**Case presentation:**

Here, we present two pediatric cases of severe and complicated typical HUS with PNS manifestations who required therapeutic treatment and an intensive multidisciplinary neuro-rehabilitation program.

In both cases, PNS manifestations were followed by the recovery from typical HUS-related severe central neurological damage and manifested mainly with marked bilateral motor deficit and hyporeflexia/areflexia in the lower limbs. The peripheral polyneuropathy was treated with immunosuppressive therapy (methylprednisolone boluses, i.v. immunoglobulins, plasma exchange), followed by a prolonged intensive neuro-rehabilitation program. After 8 months of rehabilitation, both patients gained complete functional recovery.

**Conclusions:**

PNS manifestations during typical HUS are a rare event and potentially leading to severe disability. A timely clinical assessment is mandatory to set up a prompt therapeutic and rehabilitation program and to obtain a complete clinical and functional recovery.

## Background

The hemolytic uremic syndrome (HUS) is a well-known but rare disease characterized by micro-angiopathic hemolytic anemia, thrombocytopenia, and organ damage, often renal dysfunction, which occurs both in adults and in children. The “typical” form (typical HUS) is mediated by Shiga-like toxin-producing *E. coli* (STEC) or, less commonly, by Shiga toxin-producing *S. dysenteriae* type 1 and *Streptococcus Pneumoniae* [1]. All other causes of HUS have traditionally been referred to as “atypical” (aHUS) in which main cases are due to dysregulation and over-activation of the alternative complement pathway [2–4], secondary to a gene mutation or, rarely, to acquired autoantibodies neutralizing some complement system components (e.g. anti-factor H antibodies) [5]. Rarely, clinical conditions, such as autoimmune diseases, transplantation, cancer, infectious diseases, pregnancy, or use of certain cytotoxic drugs, are associated with secondary forms of HUS [6]. In typical HUS, the Shiga-like toxin release follows the bloody diarrhea by *Entero-Hemorrhagic E. Coli*. The latter can cause systemic endothelial damage and thrombotic microangiopathy (TMA), which leads to the onset of the classic symptomatic triad (anemia, low platelet count and acute kidney injury). In severe forms of TMA with the involvement of the central nervous system (CNS), typical HUSrelated morbidity and mortality significantly increase [7]. Neurologic complications are the most common extra-renal manifestations in typical HUS, accounting 20–25% of patients. Due to these severe forms, patients are at elevated risk of the worst outcome or serious long-term disability after the acute phase of the disease [8–11]. Peripheral nervous system (PNS) manifestations during typical HUS instead is very rare and limited cases are reported [12]. At now, the key role of the complement system dysregulation is well known in the atypical HUS, while rising evidence underlies its involvement also in the pathogenesis of typical HUS [13], thus supporting the “off label” use of an anti-C5-convertase monoclonal antibody (Eculizumab) for treating more severe forms of this disease (mainly with neurological involvement) [14–17]. In this report, we describe two cases of young female patients (9 and 2-year-old), who developed a serious form of typical HUS complicated by severe CNS damage, successfully treated with Eculizumab. They were successively affected by a severely disabling peripheral neurological involvement requiring an intensive multidisciplinary neurorehabilitation program at hospital discharge.

## Case presentation

### Case 1

A previously healthy 9-years-old girl was admitted to the Pediatric Infectious Disease Unit (PIDU) with bloody diarrhea and anemia. According to regional guidelines for bloody diarrhea [18], stool research for STEC was performed and the presence of *E. Coli* O111 with gene toxin” attaching and effacing” (*eae*) was detected. On day 3, due to the rapid decline of renal function tests (serum creatinine 0,94 mg/dL, azotemia 67 mg/dL) and platelet (PLT) count (130 × 10^3^/μL) and rising of LDH (830 U/l), the patient was transferred to our Pediatric Nephrology Unit. The general conditions were poor and the laboratory parameters further worsened due to the onset of septic shock (serum creatinine 2.74 mg/dL; azotemia 125 mg/dL; WBC 31.2 × 10^3^/μL; Neutrophils 67.5%; PLT 24 × 10^3^/μL; LDH 1763 U/l; CRP 141 mg/dL). On day 5, a severe CNS involvement appeared with sudden onset of generalized paresthesia, tingling of the lower limbs, convergence of the eyes, and short but frequent absence seizures (< 1 min of duration). Despite a negative brain computed tomography (bCT) and therapy with benzodiazepines and phenobarbital, at day 6 the general conditions further worsened and a coma status arose (Glasgow Coma Score or GCS 8/15) with immediate transfer to the Pediatric Intensive Care Unit (PICU) and start of the mechanical ventilation.

The electroencephalography (EEG) revealed diffuse slow-wave activity with delta oscillations in frontal area, while brain magnetic resonance (bMR) showed diffuse ischemic lesions mainly in basal nuclei but also in the brainstem (pons, medulla oblongata). Due to severe neurological involvement, the patient received an off-label treatment with Eculizumab (300 mg i.v.) on day 6 and after a week (day 13). Moreover, despite massive i.v. fluid over-hydration and massive diuretic therapy, she became anuric and renal function tests further worsened (serum creatinine 3.3 mg/dL). For these reasons, renal replacement therapy (RRT) by veno-vein hemodiafiltration (HDF) was started at day 7 and repeated every day until day 20. The improvement of blood pressure control was achieved with amlodipine and ramipril therapy. The general condition improved rapidly, thus on day 7 the patient was extubated and returned to our Nephrology Unit. Then, the patient developed a significant visual impairment with evidence of a papilla with blurred margins and hemorrhages along the superior temporal arch in the right eye and cotton exudates at the temporal side of the left eye. These findings were compatible with bilateral retinal vascular occlusion.

Progressively, general condition and diuresis improved, with normalization of laboratory parameters (at day 25: serum creatinine 0.89 mg/dL; azotemia 49 mg/dL; WBC 7.53 × 10^3^/μL; Neutrophils 50.1%; PLT 330 × 10^3^/μL; LDH 304 U/l; CRP 5 mg/dL), so RRT was stopped. Hypokalemia resulting from brisk diuresis was treated with intravenous potassium replacement until it normalized.

**Fig. 1:**
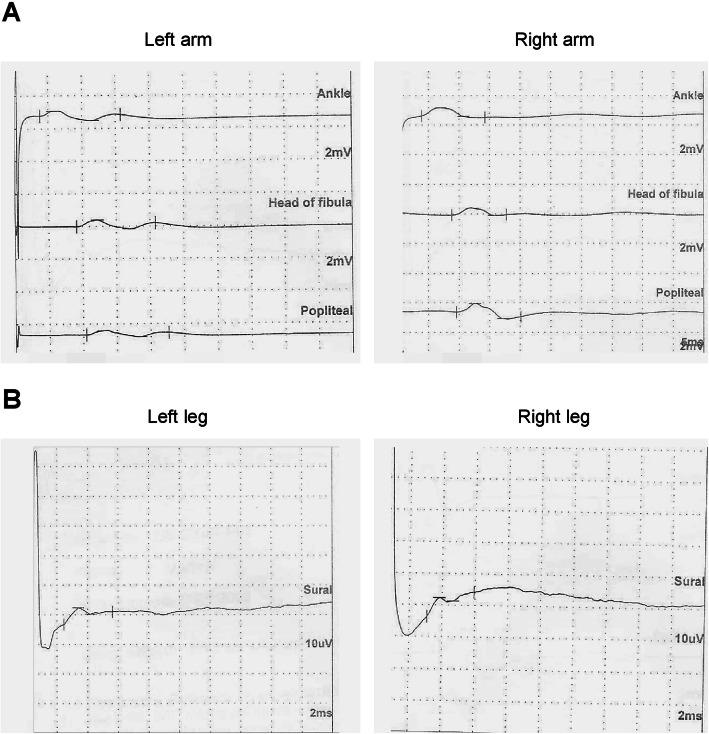
Electroneurography of peripheral nerves revealing severe polineuropathy. Low amplitude of the compound motor action potentials (CMAP) in three sites at the peroneal motor nerves (A) and reduction of sensory action potential (SAP) at sural sensory nerves, assessed bilaterally by electroneurography (ENoG) of peripheral nerves in patient 1

**Fig. 2:**
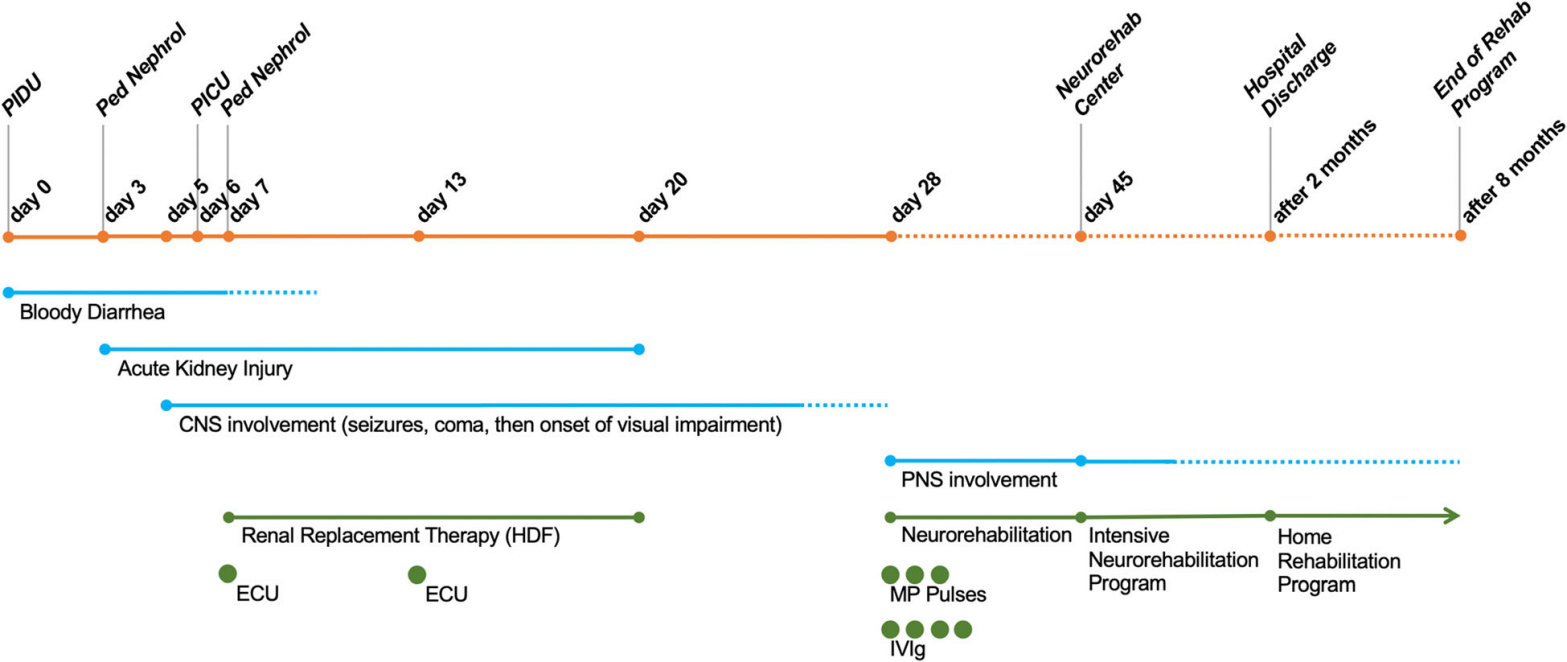
Timeline of Patient 1. CNS, central nervous system; ECU, eculizumab; IVIG, intravenous immunoglobulin; MP, methylprednisolone; PNS, peripheral nervous system

A further bMR revealed complete regression of the ischemic hypoxic areas at both the basal nuclei and the brainstem level. Also, EEG and visual impairment were normalized. On day 28, the onset of progressive intense bilateral lower limb pain with marked hyposthenia and inability to keep upright imposed further neurological evaluation. Both spinal cord MR and cerebrospinal fluid (CSF) analysis were normal. However, electroneurography (ENoG) of peripheral nerves revealed a low amplitude of compound motor action potentials (CMAP) in three sites at the peroneal motor nerve and a reduction of sensory action potential (SAP) at sural sensory nerve bilaterally, thus leading to a diagnosis of inflammatory polyneuropathy (Fig. 1A-B). Thus, combined therapy with methylprednisolone pulses (10 mg/kg × 3 days) and immunoglobulins (400 mg/kg/day for 4 days), followed by a motor-rehabilitation training program, gained a slight and progressive improvement of lower limbs pain and hyposthenia in the child. After 45 days, the patient was transferred to a neurorehabilitation center for further intensive treatment. The main clinical and therapeutic information about this case is reported as a timeline in Fig. 2.

### Case 2

A 2-years-old healthy girl was admitted to PIDU because of bloody diarrhea. Laboratory analysis showed neutrophilic leukocytosis (WBC 13,83 × 10^3^/μL, Neutrophils 67%) while other parameters were normal (Haemoglobin or HGB 13,2 g/dl, PLT 386 × 10^3^/μL, serum creatinine 0,29 mg/dl, CRP 4,8 mg/L). Fecal testing revealed enterohemorrhagic *E. coli* (STEC O111) with verocytotoxins *Stx2* and *eae*. Despite i.v. overhydration and massive diuretic therapy, she developed clinical signs of HUS with anemia, thrombocytopenia (38 × 10^3^/μL), and renal function impairment (serum creatinine 2.28 mg/dl) with worsening of general conditions and, successively, oligo-anuria. For these reasons, a session of renal replacement therapy (RRT) by HDF was started. During the first HDF session, the patient showed cardiovascular instability with the onset of neurological signs (strabismus and facial stereotypies). RRT session was suddenly stopped to perform a brain CT scan, resulted normal and then, because of worsening of metabolic acidosis (pH 7.18, HCO3^−^ 11.8 mmol/L, BE − 15), the child was moved to PICU. At admission, she showed a coma status (GCS 8/15) and required starting of mechanical ventilation. The neurological assessment revealed normal pupillary reflexes without nuchal rigidity, while EEG revealed diffuse slow-wave activity with theta-delta oscillations of low voltage.

On day 3, due to a condition of severe hyperkalemia resistant to medical therapy (i.v. calcium gluconate, i.v. sodium bicarbonate, i.v. insulin, rectal sodium polystyrene sulfonate), the patient developed life-threatening bradycardia and underwent CPR. Urgently continuous veno-vein HDF was performed over three consecutive days, then it was stopped and replaced by intermittent HDF until day 14. The onset of clinical and laboratory sign of multiorgan failure coupled to rhabdomyolysis required three sessions of CytoSorb® hemoadsorption (Cytosorbents Corporation, Monmouth Junction, NJ, USA) on day 6, 8 and 10 [19, 20].

On day 4, a brain MRI was performed, showing diffuse homogeneous signal hyperintensity in T2 and FLAIR sequences, with evident signal restriction in DWi and homogeneous hypointensity in T1-weighed sequences, localized in the deep posterior-lateral cortical area of both cerebellar hemispheres. These findings were compatible with severe post-anoxic damage, thus the patient received two doses of Eculizumab (300 mg i.v.) on day 4 and 11.

The patient remained in PICU for 14 days and was also treated with the best supportive therapy, including also i.v. antibiotics to treat a septic condition (culture-positive bronchial washing for *S. Marcescens*).

The general condition progressively improved and laboratory parameters gained almost normal values within 18 days. A further bMR imaging, on day 27, revealed a significant improvement of the ischemic hypoxic area at cerebellar level, confirmed by normal electrical activity at EEG monitoring.

After extubating, the neurological assessment revealed marked bilateral motor deficit with hyporeflexia/areflexia in the lower limbs and hyperextension of the thigh, while the upper limbs presented intentional tremors and normal reflexes. Moreover, the child also exhibited head postural tremors.

The patient returned to our Nephrology Unit on day 16. At admission, the diuresis was adequate and the renal function was recovered (serum creatinine 1.06 mg/dL). Hypokalemia resulting from brisk diuresis was treated with intravenous potassium replacement until it normalized, while blood pressure control was improved with amlodipine and ramipril.

**Fig. 3:**
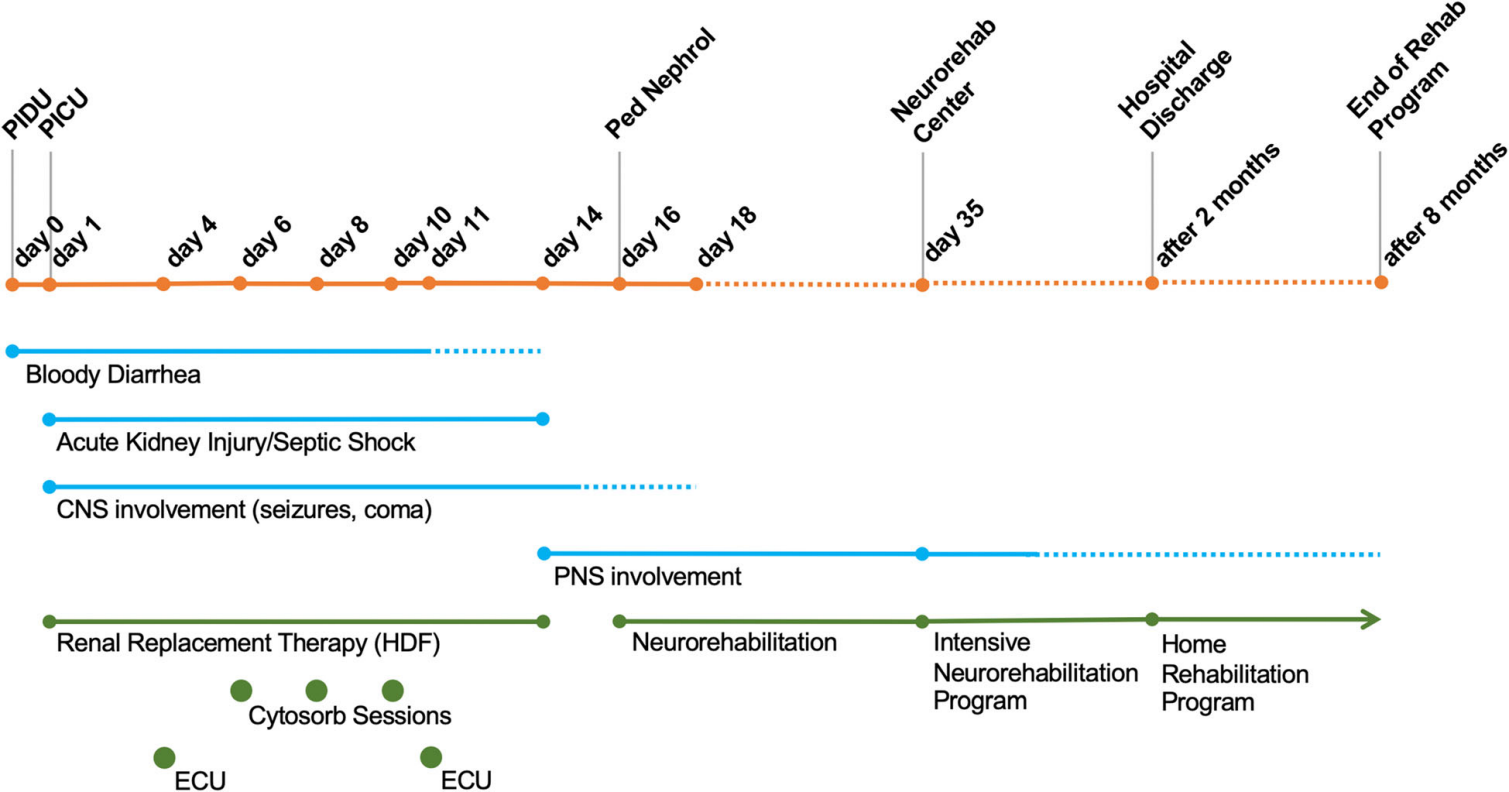
Timeline of Patient 1. CNS, central nervous system; ECU, eculizumab; PNS, peripheral nervous system; TPE, Therapeutic Plasma Exchange

Due to the persistent severe bilateral motor deficit with hyporeflexia/areflexia in the lower limbs and intentional tremors of both the upper limbs, a deep neurological reassessment with ENoG revealed severe axonal damage with both motor and sensory demyelination of the lower limbs, mainly at right. The peripheral polyneuropathy was treated with therapeutic plasma exchange (TPE) (seven treatments), but the functional recovery was unsatisfactory, because of the persisting of lower limb asthenia and bilateral absence of osteotendinous reflexes. Thus, on day 35 the patient was discharged and was transferred to a neuro-rehabilitation center for intensive treatment. Main clinical and therapeutic information about this case is reported as a timeline in Fig. 3.

### Neuro-rehabilitation program

Before starting the treatment, neurological examination and physiatric functional evaluation in both patients revealed an inability to walk and symmetrical hypotonia, with lower limbs weaker than upper limbs, lying in a frog-like position, deep tendon reflexes decreased in all extremities. No Babinski’s bilateral signs were present. Furthermore, patient 1 showed distal lower limbs mild hypotrophy and scoliosis, while patient 2 clinical presentation was more severe, with bilateral ankle contractures. The neuropsychological profile was within normal limits in both patients.

In patient 1, visual evoked potential showed both the reductions in P100 amplitude and increments in P100 latency at the right occipital area, while no specific abnormalities have been revealed on the left. The latter was normal in patient 2, who instead presented retinal dystrophy in the right eye and scotopic and photopic full-field ERG characterized by reduced amplitudes particularly in scotopic condition; the left eye was normal.

In these cases, physiotherapy and occupational therapy were started including specific therapeutic activities; during the first stage, when the voluntary movement was compromised, passive kinesitherapy has been initiated to improve local blood circulation and to avoid joint stiffness, tendon retractions, and muscle contractures due to protracted immobility.

After the appearance of voluntary activity event, active kinesitherapy has been progressively introduced through a gentle submaximal and aerobic functional exercise, that was carried out with adequate monitoring, with pauses according to the needs of the patient and with graduated assistance [21].

In patient 2, ankle foot orthoses (AFOs) was also used to minimize contractures and to improve both the stance and swing phases of gait.

Moreover, the neuro-rehabilitation program included exercises to improve coordination and balance. Our patients received this specific and intensive physiotherapy program and, after a 2 months term, they developed a progressive improvement on muscular strength and on motor functions.

We have used the Functional Independence Measure for Children (WeeFIM) [22] to assess outcomes from neuro-rehabilitation and significant changes have been found in both patients particularly concerning to the motor scale (Table 1).

**Table 1:** Overview of the Functional Independence Measure (FIM) for children at admission and discharge

Case 1			Case 2		
***Item (scale)***	***Admission***	***Discharge***	***Item (scale)***	***Admission***	***Discharge***
FIM motor	2.08	4.62	FIM motor	1.23	1.69
FIM cognitive	3.22	5.11	FIM cognitive	2.6	2.6
FIM total	58	92	FIM total	29	35

In both patients, neurological examination revealed the recovery of autonomous deambulation and improvement in postural changes, but the walk was still unstable, with a wide base and the feet thrown out, and the equilibrium was mildly unstable. Besides, both children showed full adherence to the hospital-based neuro-rehabilitation program without significant adverse events, and they continued a home-based rehabilitation once discharged. After further 8 months of home rehabilitation, both patients were re-evaluated with evidence of complete functional recovery.

### Genetic screening

Both the patients were clearly affected by a STEC-HUS related to *E. Coli* O111 infection. However, due to very severe neurological impairment, genetic screening of all exons and flanking regions of CFH, CD46, CFI, CFB, C3, THBD, DGKE, C5, CFHR1–5, MMACHC and ADAMTS13 was performed through next-generation sequencing (NGS), as described elsewhere [23, 24], but no pathogenic variants were identified. Moreover, none of them presented genetic variants of C5 gene, which are related to low response to Eculizumab [25].

## Discussion and conclusion

Neurological involvement is the most frequent extra-renal manifestation during typical HUS, accounting for almost 17–34% of patients [7]. This complication significantly worsens patients’ survival and may lead to severe permanent disability, since end stage renal disease requiring chronic hemodialysis and/or kidney transplantation following typical HUS-induced AKI accounts for only 3% of cases [26–28].

Typical HUS-mediated CNS injury may vary from mild to severe and potentially life-threatening manifestations, ranging from eye disorders (strabismus, nystagmus, visus disorders/amaurosis), to alterations of communication skills, to CNS disorders (hypo/hypertensive disorders of muscle tone, neuro-vegetative system disorders, alteration of consciousness, epileptic seizures until coma) [7].

Among neurological assessment techniques, brain MR is actually the gold standard method to analyze brain damage location and extent during typical HUS, which mainly, but not only, involves basal nuclei with a pattern in early diffusion-weighted T1 sequence. However, given the wide clinical spectrum of neurological signs, lesions at different levels of CNS have also been reported [29]. Based on both MR findings and the location of the lesions, the main pathogenic mechanism seems to be ischemic injury after thrombotic microangiopathy onset with rising evidence of complement system involvement [9]. Although controlled clinical trials are lacking, all these findings strongly suggest a key role of early administration of Eculizumab, and anti-C5 convertase monoclonal antibody, to halt and potentially reverse the TMA-mediated severe neurological involvement, as well as at renal levels. Rising evidence support the efficacy and safety of Eculizumab administration to treat the severe neurologic involvement in typical HUS patients [30, 31]. Indeed, even in our cases, the central nervous system damage following typical HUS onset was effectively reverted by early Eculizumab administration.

Although several works describe a wide spectrum of clinical and radiological signs affecting CNS during typical HUS, very little is reported about peripheral nerve involvement. In a single report, the authors described the case of a pediatric patient with severe STEC-HUS belatedly complicated by a disabling peripheral neuropathy, as in our two cases [12].

Neurological emergencies are frequent in patients with hematological disorders, for instance during thrombotic microangiopathies, which involves both the central and peripheral nervous system [32]. In limited cases, when nerve biopsy was available, thrombosis of epineurial blood vessels with inflammation was shown, thus demonstrating that thrombotic microangiopathy can involve the peripheral nerves, resulting in major morbidity [33]. In this report, we described two cases of severe PNS involvement following typical HUS and underline the efficacy of a prompt neuro-rehabilitation program, coupled with adequate supportive care. The pathogenic mechanism of peripheral nerve involvement during typical HUS is unknown. In experimental models, the evidence of verotoxin receptors in peripheral nerves strongly suggests a possible direct neuropathic effect of STEC infection [34]. However, in our two cases, the lack of nerve biopsy does not allow us to draw definitive conclusions about the pathogenesis of the typical HUS-related peripheral neuropathy. Intriguingly in both cases, the late onset of PNS involvement after resolution of CNS injury seems to suggest a possible role for dystrophic phenomena affecting the peripheral nerves, probably due to typical HUS-related lack of nutrients (i.e. probiotics or vitamins) [35], which in other clinical settings may lead to peripheral neuropathy [36, 37]. Generally, we can consider the peripheral nerve as a complex system in which the endoneurial homeostasis is strictly regulated via tight junction-forming endoneurial microvessels that control ion, solute, water, nutrient, macromolecule, and leukocyte influx and efflux between the bloodstream and endoneurium; thus, it is not daring to speculate that the STEC-related blood stream disorders (i.e. leukocytosis, electrolyte alterations, toxin trafficking) may affect the blood-nerve barrier integrity and function, thus leading to more or less severe decay of the peripheral nervous system [38].

A correct diagnostic evaluation could help us to differentiate several types of muscular disorders, although the temporal sequence of clinical events suggests a close correlation between HUS and PNS manifestations [39]. Furthermore, pediatric neuromuscular diseases involving PNS are mainly due to genetic etiology, and therefore they can easily be ruled out in our cases which have occurred as a result of an acute illness [40].

Although immunotherapies with monoclonal antibodies may induce peripheral neuropathic side effects and, rarely, immune axonal acquired neuropathies [41], this complication seems unlikely after Eculizumab infusion and up to now no cases have been described. Moreover, in the second case here reported, the severe peripheral neuropathy could be immune-mediated, thus suggesting a rescue therapy with plasma-exchange (PE). Unlike rehabilitation therapy, this approach did not gain any benefit and has been halted after 6 sessions. The absent response to TPE further excludes the hypothesis of an immune-mediated peripheral neurological damage in our cases.

In conclusion, peripheral nerve involvement during typical HUS is a very rare, but potentially severely invalidating event. Although we described just two cases of severe PNS involvement following typical HUS infection, the early treatment of this complication with prompt neuro-rehabilitation, coupled with adequate supportive care, is essential to prevent permanent and irreversible damage and may significantly improve the clinical outcome of these pediatric patients.

## Data Availability

The datasets used and/or analysed during the current study are available from the corresponding author on reasonable request.

## Abbreviations

AFO: Ankle Foot Orthoses
aHUS: atypical Hemolytic uremic syndrome
BE: Base Excess
CFE: Cerebrospinal fluid examination
CMAP: Compound Motor Action Potentials
CNS: Central nervous system
CPR: Cardiopulmonary resuscitation
CRP: C-reactive protein
CT: Computer Tomography
EEG: Electroencephalography
EHEC: Entero-Hemorragic *Escherichia Coli*
EMG: Electromyography
ENoG: Electroneurography
ERG: Electroretinography
FIM: Functional Independence Measure
GCS: Glascow Coma Score
HCO^3−^: Bicarbonate Ion
HDF: Hemodialfiltration
HGB: Haemoglobin
HUS: Hemolytic uremic syndrome
IV: Intra-venous (therapy)
LDH: Lactate dehydrogenase
MR: Magnetic resonance
Neut: Neutrophils
PICU: Pediatric intensive care unit
PLT: Platelets (count)
PNS: Peripheral nervous system
RRT: Renal replacement therapy
SAP: Sensory Action Potential
sCr: serum Creatinine
STEC: Shiga-like toxin-producing *E. coli*
TMA: Thrombotic Micro-Angiopathy
TPE: Therapeutic Plasma Exchange
WBC: White blood cell (count)
